# Blended e-learning and certification for medicines development professionals: results of a 7-year collaboration between King’s College, London and the GMDP Academy, New York

**DOI:** 10.3389/fphar.2024.1417036

**Published:** 2024-06-19

**Authors:** Honorio Silva, Peter Stonier, Pravin Chopra, Jacob Coots, Domenico Criscuolo, Soneil Guptha, Stuart Jones, Sandor Kerpel-Fronius, Gustavo Kesselring, Xavier Luria, David Morgan, Eddie Power, Sam Salek, Gustavo Silva, Tamas Suto, Kamlesh Thakker, Pol Vandenbroucke

**Affiliations:** ^1^ GMDP Academy, The Academy of Global Medicines Development Professionals, New York, NY, United States; ^2^ Faculty of Life Sciences and Medicine, King’s College London, London, United Kingdom; ^3^ Center for Pharmaceutical Medicine Research, King’s College London, London, United Kingdom; ^4^ Genovax Pharma, Milan, Italy; ^5^ Semmelweis University, Budapest, Hungary; ^6^ PharmaTrain Federation, Brussels, Belgium; ^7^ School of Life and Medical Sciences, University of Hertfordshire, Hartfield, United Kingdom; ^8^ Merck Group, Eysins, Switzerland

**Keywords:** certification, medicines development, pharmaceutical medicine, e-learning, King’s, life-long learning, continuing professional development

## Abstract

**Introduction:**

The field of Medicines Development faces a continuous need for educational evolution to match the interdisciplinary and global nature of the pharmaceutical industry. This paper discusses the outcomes of a 7-year collaboration between King’s College London and the Global Medicines Development Professionals (GMDP) Academy, which aimed to address this need through a blended e-learning program.

**Methods:**

The collaboration developed a comprehensive curriculum based on the PharmaTrain syllabus, delivered through a combination of asynchronous and synchronous e-learning methods. The program targeted a diverse range of professionals serving in areas related to Medical Affairs.

**Results:**

Over seven annual cohorts, 682 participants from eighty-six countries were enrolled in the program. The program’s effectiveness was assessed using Kirkpatrick’s model, showing elevated levels of satisfaction (over 4.0 on a five-point scale), suggesting significant gains in competence at the cognitive level and leveraged performance. Notably, 70% of responding alumni reported significant improvement in their functions, corroborated by 30% of their supervisors. The further long-term impact of the program on their respective organization has not been established.

**Discussion:**

The GMDP Academy’s program has significantly contributed to life-long learning in Medicines Development, addressing educational gaps and fostering interdisciplinary collaboration. Its success highlights the importance of continuous education in keeping pace with the industry’s evolving demands and underscores the potential of blended learning in achieving educational objectives in pharmaceutical medicine.

## Introduction

### Background: from discipline to profession

The Medicines Development Workforce has grown to be a multidisciplinary interprofessional global body of qualified professionals working around the pharmaceutical industry, academic institutions, and research sites.

For over 40 years Pharmaceutical Medicine was conceived as a medical scientific discipline for the discovery, development, evaluation, registration, monitoring, and medical marketing of medicines for the benefit of patients and community health (Stonier et al., 2007; Faculty of Pharmaceutical Medicine, 2024). The aim to be positioned as a medical specialty motivated the creation of national professional associations organized under the umbrella of IFAPP (International Federation of Pharmaceutical Physicians and Pharmaceutical Medicine (IFAPP, 2024) in 1975. Postgraduate education programs were established in the UK, pioneered by Cardiff University (Salek and Luskombe, 1994) followed by others in Europe, although restricted only to physicians. However, the recognition of Pharmaceutical Medicine as a medical specialty was achieved only in four countries (UK, Ireland, Switzerland, and Belgium).

In parallel, non-medically qualified professionals -referred as medicines development scientists (MDS)- from health-related disciplines have gradually taken the traditional medical related roles within pharmaceutical organizations, regulatory agencies, and contract research organizations and today physicians and non-physicians lead drug development groups. This change relates to the advanced scientific developments due to the integration of systems biology and Omics in research and development.

As a result, Pharmacogenomics and personalized medicine, translational research, data driven research, bioinformatics, computational modelling, digital technologies, and artificial intelligence have emerged as nascent disciplines in medicines development. Contemporary ethical and legal aspects, patient centered research as well as clinicians’ engagement are now included as part of ongoing discussions. Consequently, modern nomenclature has been proposed to define new disciplines within the profession.

Medical Affairs (MA) has also emerged as a strong third pillar between traditional clinical development and the commercial groups in pharmaceuticals. MA groups, including PP and MDS, aimed to bridge this gap, have shown the largest growth in size and scope of responsibility in the last decade (Evers et al., 2019; Galateanu et al., 2019; Shapard et al., 2024).

Pharmaceutical Medicine has thus grown to encompass global dimensions including thousands of non-medically qualified scientists (MDS) beyond physicians and incorporating the above mentioned emerging scientific disciplines. The terms “Medicines Development” or “Medicines Development Sciences” have been accepted as an equivalent or synonymous term. (PharmaTrain, 2024).

The practice of Medicines Development and its related disciplines meet the criteria for denomination as a distinct profession in health sciences (Otterley, 2018; Gardner and Shulman, 2005; Brante, T, 2011; Australian Council of Professions, 2003). However, the awareness of either the discipline or the profession is limited among key stakeholders.

### Educational needs and resources

The mismatch between the profiles of the graduates from academic programs on disciplines on health-related arenas, including those related to medicines development, and the changing needs of the various stakeholders (pharmaceutical industry, regulatory agencies, contract research organizations, universities) as well as the various healthcare systems around the world, suggests that traditional education programs cannot fill the gap (Frenk, J et al., 2010). Thus, a redesign of professional education has become necessary, aiming for transformative learning and interdependency in education (Harden, RM, 2007; Barber, M et al., 2013; Drago, D et al., 2016; UK General Medical Council, 2009). Professional Competencies and Capabilities in Pharmaceutical Medicine have been developed (Silva et al., 2013; Stonier et al., 2018; Faculty of Pharmaceutical Medicine, 2020) to serve as a resource and guide for those interested in improving the quality and accountability of medicines development graduates and lifelong learning programs.

The PharmaTrain Federation is a not-for-profit organization that started its activities as an IMI (Innovative Medicines Initiative) European Project. Its mission is to drive implementation of globally recognized high-level standards for postgraduate education and training in Medicines Development. The PharmaTrain syllabus (prepared as joint effort with IFAPP and the Faculty of Pharmaceutical Medicine, 2023) defined the standards for education and training in Pharmaceutical Medicine/Medicines Development. (PharmaTrain, 2018; 2024).

This syllabus, based on a set of required *Learning Outcomes* (LO) for such level of postgraduate education and training, has become an integral part of the curriculum for graduate and lifelong learning programs for most disciplines involved in medicines development. The LO are statements of what a student is expected to know, understand and/or be able to demonstrate after completion of a process of learning. The LOs of the PharmaTrain syllabus are aligned to the IFAPP-PharmaTrain competencies (Stonier P, et al., 2020).

However, nowadays a significant percentage of the professionals that work in this complex environment are trained on-the-job. Experiential learning can increase productivity and efficiency in specific industries and can benefit the company, from reducing training costs to creating more effective employees, but this is typically only achieved in structured, on-the-job training such as apprenticeships (Department of Business Innovation and Skills, 2012). Without a structured, externally accredited system, employees can become experts in silos within the development process, and this may eventually be a hindering factor for individual professional growth, as well as for the development of a cadre of senior executives who can manage this complex process across disciplines.

Evidence for the scale of the educational problem in medicines development has been gathered through surveys conducted among members of 28 national members associations affiliated to IFAPP, which showed that only 20% of the membership had received formal postgraduate education in medicines development (Silva H, et al., 2012).

Several experiences have been published pointing to the global need for education and training among individuals working in the various arenas of medicines development as well as in clinical research sites (Stonier P, 2011; Silva H, et al., 2015; 2013). More recently our group conducted a survey in four countries where recognized courses for education in pharmaceutical medicine are established: Japan, Italy, Spain, and Brazil. Combined results were analyzed in common demographic profiles. Most respondents were working in the industry, with over 10 years of experience. When compared across seven (7) domains of competencies, variable levels of competence and significance to their position were observed for all participants pointing out the need for global training in medicines development. (Imamura K, et al., 2019).

The time was ripe for an initiative aligned between the pharmaceutical industry, academic institutions and learned professional associations to provide competency-based education through lifelong learning programs in the professional training of medicines developers working in the Medical Affairs arena.

### Planning the educational intervention

In 2016, the IFAPP Academy was created to meet the need of several healthcare companies operating around the world to provide education and training in addition to professional certification as well as other training and professional development courses in the areas of Medical Affairs and Medicines Development. The Academy designed and implemented an e-Learning program in partnership with IFAPP (International Federation of Associations of Pharmaceutical Physicians and Pharmaceutical Medicine), with the shared goal of fostering continuing professional education.

As mentioned above, several factors such as the evolving healthcare stakeholder landscape, more empowered patient advocacy, and advancing digital medicine interfaces, with increasing demand for diverse skill sets, encompassing the spectrum of technical and business acumen*,* resulted in a revised role for the Medical Affairs function within pharmaceutical companies and contract research organizations (CROs) and the need for education and further training.

The newly formed IFAPP Academy also entered a strategic alliance with King’s College London immediately after its incorporation as a nonprofit organization in the USA. King’s College London, through its Center for Pharmaceutical Medicine Research, which is a PharmaTrain accredited Centre of Excellence, has been a key partner of the Academy from its inception. In addition to providing expert faculty, King’s has appointed representatives participating in the governance and quality assurance of the annual curriculum and its delivery which executed the alignment of the discipline of pharmaceutical medicine with the training in medicines development. Regular meetings have been held throughout to discuss the program and its outcomes.

Four major pharmaceutical companies (Pfizer, Sanofi, Bayer, and Merck) provided the seed funds to start the program. Other pharmaceutical companies joined as sponsors, although with a lower cohort size. As a result, a 10-month on-line Certification program consisting of six modules with focus in Medical Affairs in Medicines Development was launched in 2017. Almost 700 students have received the award certification to date. In addition to the company sponsored students, a gradual increase in the number of self-sponsored students has been observed.

In 2019 a strategic collaboration was developed with the Tufts University Center for Drug Development Studies (Tufts CSDD) to produce an online program on “Leadership in Medicines Development” also run on an annual basis. The outcomes of this program will be included in a separate report.

After 7 years of successful operation and considering the gradual increase of non-medically qualified professionals joining medicines development and medical affairs teams within the pharmaceutical industry, the opportunity arose to move forward in a new direction, objectives, vision, and mission. The Academy Board decided to modify, among other changes, the name of the entity, the organizational structure, the business model, and its deliverables, assuming as of 2023, the new global name as *GMDP Academy (The Global Medicines Development Professionals Academy)*. The overall intent was to align the program to foster PM/MD as a profession, encompassing physicians and MDS. The value proposition is that professional success could be achieved through proper education and training, supported by professional identity and a clear individual purpose.

### Outcomes: the 7-year experience

The program provides the IFAPP-Pharma Train competencies (at the cognitive level) to health professionals involved in Medical Affairs and related functions, a growing area in Medicines Development within the pharmaceutical industry. Its syllabus is adapted from PharmaTrain’s, and the curriculum includes 64 online asynchronous lectures, organized around six modules, including a total of 24 synchronous webinars, 25 discussion forums, weekly student consultations, regular evaluations, end-of-module discussion sessions on various subjects and a comprehensive end-of-program assessment, involving essays, using an interactive approach during a 10-month period. (https://gmdpacademy.org/certification-programs/certification-in-medicines-development/).

Students are assigned to 5–10 sub-groups and are invited to make an online synchronous presentation on a hot topic in Medicines Development at the end of each module, assessed under standardized criteria. This is aimed to foster team building and working in collaboration throughout various regions and companies.

Sponsors receive regular reports on the progress of their respective sponsored annual cohorts and their overall assessment outcomes are discussed with company representatives.

A Certificate of Attendance offered by the King’s College London and a Professional Certification granted by the GMDP Academy is offered to those students successfully completing the program.

### Assessment: methods and results

A total of 682 students from 86 countries participated in the educational offerings during the period 2017–2023. The program effectiveness was assessed using the traditional Kirkpatrick’s four levels of training evaluation (Kirkpatrick, 1954; Kayser J and Kayser W, 2016), which includes Reaction, Learning, Behavior and Organizational Results.

**Level 1: Reaction** (what the participants thought at the end of the program) was assessed using a one-to-five Likert visual analogue scale, which showed elevated levels of satisfaction (overall mean response >4.0) among all seven cohorts.

**FIGURE 1:**
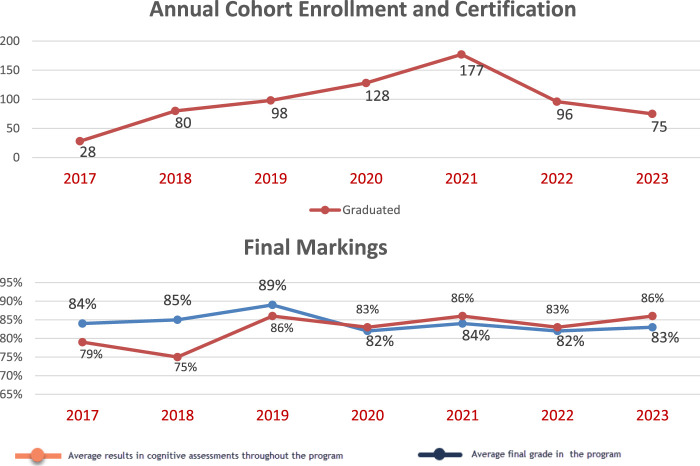
Annual cohort enrollment and certification + final markings.

**Level 2: Learning** or gain in scientific knowledge, was assessed through the integrated examinations, at the end of each of the six modules and the final assessment. The threshold marking to pass the End of Module Assessments and all activities included in the Program was set at 66% and 60%, respectively. This was significantly exceeded throughout the seven cohorts. The mean annual success rate was 83% (Figure 1).

**Level 3: Changes in job behavior** resulting from the program, aimed to identify whether the learning has been applied, was assessed through an online survey conducted among the alumni. The questionnaire, created and revised by the GMDP Academy Steering Committee and approved by the Board, included 10 items focused on self-reported performance improvement and one open-ended question related to suggestions for program leverage (summary in Table 1). Each question was formulated using an ordinal scale (1 = no improvement; 2 = some improvement; 3 = noticeable improvement; 4 = significant improvement; 5 = major improvement recognized by supervisor).

**TABLE 1:** Assessment of performance (Kirkpatrick level 3) N = 64 responses/405 valid invitations/682 total alumni. Assessment of Program Impact among alumni using a Kirkpatrick scale adapted questionnaire.

Question	Total positive* (%) and (SE** %)	Notes
How confident do you feel in implementing the approaches to problem solving and skills taught in the Academy’s programs	**86 (4)**	
How relevant do you find the training content in the Academy’s program to your specific job roles and responsibilities?	**83 (5)**	**60% responded as “Highly relevant” or “Extremely relevant”**
Have you noticed an improvement in your work performance since completing the Academy’s training programs?	**70 (6)**	**32% responded “Major improvement recognized by my supervisor and stakeholders.”**
Have you recommended the Academy’s program among your colleagues and/or outside of your organization?	**87 (4)**	

^a^ Total positive defined as proportion of subjects answering 3–5 on the ordinal scale, e.g., Often/Very Often/Always OR, Noticeable Improvement/Significant Improvement/Major Improvement.

^b^ SE: standard error.

The process of survey posting, invitations, distribution, data collection and reminder notifications, was executed via Survey Monkey. (www.surveymonkey.com). The survey was not anonymous.

601 of the 682 alumni of the course were contacted at their last known email address. 137 addresses were no longer active. Of 464 alumni reached, 7 declined to complete the survey and 393 failed to respond, despite email reminders. This resulted in 405 valid invitations, from which 64 responses (15.8%) from alumni based in all continents, were received. The initial invitation was sent on 18 December 2023. Follow-up reminders were sent on 23 December 2023, and 2 February 2024. The reminders were targeted only at those who had not responded or had only partially responded to the initial survey.

The results were very encouraging. Alumni reported a positive response (defined as a score of 3 or over) ranging from 70%–86% to the posted questions, particularly as related to performance improvement and change of attitudes. Furthermore, 87.5% recommended the program to their colleagues. With a sample size of 64, the standard error of each of the estimated proportions is around 5% (Table 1).

**Level 4: Organizational Results**. While this is probably the most difficult level of evaluation, it was not intended as part of the objectives of our program, Annual surveys using self-administered questionnaires conducted at one of the participants’ organisations consistently demonstrated increased knowledge transfer and behavioural change among participants of the Academy course. In addition, enhanced organisational impact was also reported by the participants’ managers. Furthermore, we observed a positive correlation between participation in the course and improved annual performance and career progression (data on file) at this organisation. However, these results should be interpreted as anecdotal at this time.

Since this was not a vocational program, no specific skill gains were assessed. Our evaluation focused on the cognitive aspects of the competencies, self-reported changes in attitudes and performance improvement.

## Limitations

The 64 responses represent only 16% of the 405 invitations known to have reached alumni, which itself is around 60% of those who have passed through the course. While disappointing, this is not an unusual figure for social science research (Holtom et al., 2022). Clearly the performance assessments could be subject to bias if the responders tended to be students with more positive or more negative views than the population in general. This cannot be known with certainty, but review of the responder sample shows no systematic differences from the population as a whole: all regions of the world and all cohorts were represented in the program.

## Discussion

In the last few decades, the discipline of pharmaceutical medicine has changed drastically. It has moved from a discipline only for physicians, who functioned as single champions of a medicine development plan, to encompass all members of the medicines development process. This has enabled patients, medicines quality and safety to be the center of the pharmaceutical medicine training syllabus, which is taught to a wide range of stakeholders, including patients themselves (Haerry et al., 2018).

However, despite these changes and having the capability to solve the medicines development training problems there is still a lack of formal recognition of the situation and device potential solutions. Therefore, no mandatory qualifications are required for physicians and scientists entering the world of medicines development, either joining a pharmaceutical company or a Contract Research Organization.

The GMDP Academy course in Medicines Development was developed in response to the growing educational gap and it has achieved a significant success, demonstrated by the participation of almost 700 students from 86 countries and the outcome metrics.

The program syllabus has undergone continuous revision to reflect the advances in the various disciplines involved in medicines development during this period. This coupled with students’ feedback and annual review of individual modules has ensured continuous improvement of the program, meeting the education and training needs of the students and their employers.

Additional educational programs and a professional career path, including multiple certifications and customized offerings, are currently under consideration, to ensure competent professionals are adopting pharmaceutical medicine as their professional discipline, attaining a sense of purpose, and contributing to bringing better medicines to meet patient and societal needs.

The collaboration with King’s College London: the GMDP Academy program is unique in having established a close collaboration between academics based in the discipline of pharmaceutical medicine and professionals with experience in medicines development. This collaboration guarantees the academic integrity of the evaluation of the learning outcomes, as well as lecture material that is fully up to date with the most recent developments in the field and quality seal of approval. Together, they underpin the value of the recognition offered to the students who successfully complete the program.

The students’ commitment is another important cornerstone for success. Before the start of each course, a “learning agreement” is submitted to all students. The mandatory assignments are clearly laid out: hours per week to dedicate to lectures, study of the bibliography, presence at the online webinars, participation in working groups and finally the elaboration of two End of Program Assessments. A clear message to students is important to make them aware of the prominent level of quality expected.

Finally, the regular availability of all faculty in a “Discussion Forum” could help students to clarify queries in the lectures, or to ask for additional tutoring. Indeed, even though our course is web-based, the number of interactions among students in the working groups, and the possibility to address questions to all teachers make it a very lively experience.

The outcomes of our program after the use of the appropriate tools confirm the value of online education on Health Professions Education, as applied to the Medicines Development Sciences. (Masters K, et al., 2024). However, we should acknowledge the limitations of our assessment, as described above, and the emergence of other assessment methods (Tamkin P, et al., 2002) which could be used as additional tools for the evaluation of educational programs.

## Conclusion

The GMDP Academy course started 7 years ago, a period long enough to draw tentative conclusions, considering the above-mentioned limitations. Our students valued the course which not only gave them a comprehensive vision of the Medicines Development process, but also helped them to grow professionally and make significant changes in their approach to and involvement in Medicines Development. In many cases it was also allowed for meaningful career progression as well as being instrumental for the assignment of a new job, with greater responsibilities. One of the program goals, which is to help our students to advance in their professional career, has been met. Importantly, the course made students aware of their identity and purpose as professionals in medicines development.

This is an exciting time for the pharmaceutical industry to capitalize on technological advances which provide opportunities for sharp-edge innovation and developing medicines for diseases of the 21st century and for an increasingly aging population. We are entering the digital era and a rapid growth of artificial intelligence as well as immense potential of digital medicine delivery. The consequences of such exciting technologies would be reflected in the curriculum and educational tools, demanding a rapid adaptation in the approaches to lifelong learning. Anticipating changes is key to maintaining success.

Finally, we believe that our commitment to the generation of a high-quality course in Medicines Development has and would continue to help professionals in Pharmaceutical Companies, CROs, Regulatory Authorities, and hospital-based Clinical Trial Centers to bring better medicines to patients, carers and provide benefit to society.
